# mTORC1 suppresses PIM3 expression via miR-33 encoded by the SREBP loci

**DOI:** 10.1038/s41598-017-16398-y

**Published:** 2017-11-23

**Authors:** Ilana Kelsey, Marie Zbinden, Vanessa Byles, Margaret Torrence, Brendan D. Manning

**Affiliations:** Department of Genetics and Complex Diseases, Harvard T.H. Chan School of Public Health, Boston, MA USA

## Abstract

The mechanistic target of rapamycin complex 1 (mTORC1) is a central regulator of cell growth that is often aberrantly activated in cancer. However, mTORC1 inhibitors, such as rapamycin, have limited effectiveness as single agent cancer therapies, with feedback mechanisms inherent to the signaling network thought to diminish the anti-tumor effects of mTORC1 inhibition. Here, we identify the protein kinase and proto-oncogene PIM3 as being repressed downstream of mTORC1 signaling. PIM3 expression is suppressed in cells with loss of the tuberous sclerosis complex (TSC) tumor suppressors, which exhibit growth factor-independent activation of mTORC1, and in the mouse liver upon feeding-induced activation of mTORC1. Inhibition of mTORC1 with rapamycin induces PIM3 transcript and protein levels in a variety of settings. Suppression of PIM3 involves the sterol regulatory element-binding (SREBP) transcription factors SREBP1 and 2, whose activation and mRNA expression are stimulated by mTORC1 signaling. We find that PIM3 repression is mediated by miR-33, an intronic microRNA encoded within the SREBP loci, the expression of which is decreased with rapamycin. These results demonstrate that PIM3 is induced upon mTORC1 inhibition, with potential implications for the effects of mTORC1 inhibitors in TSC, cancers, and the many other disease settings influenced by aberrant mTORC1 signaling.

## Introduction

Due to its positioning at a critical nexus between upstream growth signals and downstream anabolic processes, the conserved serine/threonine protein kinase complex mechanistic target of rapamycin complex 1 (mTORC1) is a key driver of cell growth, including the uncontrolled growth of tumor cells. mTORC1 is frequently activated in human cancers, across nearly all lineages, and would seem to be a prime target for precision therapies^1^. With few exceptions, however, mTOR-targeted therapies alone have proven insufficient to cause tumor regression, in part due to the complexity of the mTORC1 signaling network, among other reasons^2,3^. While upstream inputs into mTORC1 signaling and mTORC1-mediated control of anabolic processes downstream have been extensively characterized^4,5^, less is understood about effectors whose activity is repressed by mTORC1 signaling, and the role these effectors might play in the response to pharmacological inhibition of mTORC1.

As a key regulator of cell growth and metabolism, mTORC1 is situated downstream of several major pathways involved in the sensing of growth factors, cellular energy levels, and nutrient availability, including the PI3K-Akt, Ras-Erk, and AMPK pathways^4^. Aberrant activation of mTORC1 signaling in cancer is primarily due to the frequent misregulation of these upstream signaling pathways, which converge to regulate the TSC protein complex (TSC1-TSC2-TBC1D7), a key negative regulator of mTORC1^2^. Inactivating mutations in the TSC complex or direct inhibitory phosphorylation from upstream oncogenic pathways cause constitutive activation of mTORC1^6–11^. This activation enables mTORC1 to promote its downstream processes, including protein, nucleotide, and lipid synthesis^12–18^. While there have been extensive studies to characterize the upstream regulation of mTORC1^19^, we are only beginning to fully understand the scope of the downstream consequences of mTORC1 activation.

A variety of omics approaches have been employed to define the downstream functional repertoire of mTORC1 signaling, including transcriptional profiling, ribosomal profiling, phospho-proteomics, and metabolomics^13,15–18,20–22^. Genetic settings with loss of the TSC tumor suppressors, leading to constitutively active mTORC1 signaling, together with the use of mTORC1 inhibitors such as rapamycin, have been particularly powerful in expanding our knowledge of mTORC1 functions and crosstalk regulation with other cellular pathways and processes. Here, we use such an approach to identify the proto-oncogene PIM3 as a downstream target inhibited by mTORC1 signaling. Using TSC-deficient mouse embryonic fibroblasts (MEFs), we show that PIM3 inhibition is coupled to mTORC1 signaling via the transcription factors SREBP1 and 2 (sterol regulatory element-binding proteins 1 and 2). SREBP transcriptional activity is induced by mTORC1 via its stimulation of SREBP processing from an endoplasmic reticulum-bound inactive form to a mature nuclear form^18,23–26^. The mTORC1-mediated increase in SREBP transcriptional activity induces the transcript levels of its own gene products *Srebf1* and *Srebf* *2* ^18^, and we find that this regulation causes a concomitant increase in the levels of its intronic microRNA, miR-33^27^. We demonstrate that induction of miR-33 downstream of mTORC1 suppresses PIM3 expression. These findings highlight a previously unappreciated mechanism of gene repression downstream of mTORC1, and demonstrate an unexpected outcome of treatment with mTORC1 inhibitors in the induction of the PIM3 proto-oncogene.

## Results

### PIM3 expression is repressed downstream of mTORC1 and induced by mTOR inhibitors

**Figure 1:**
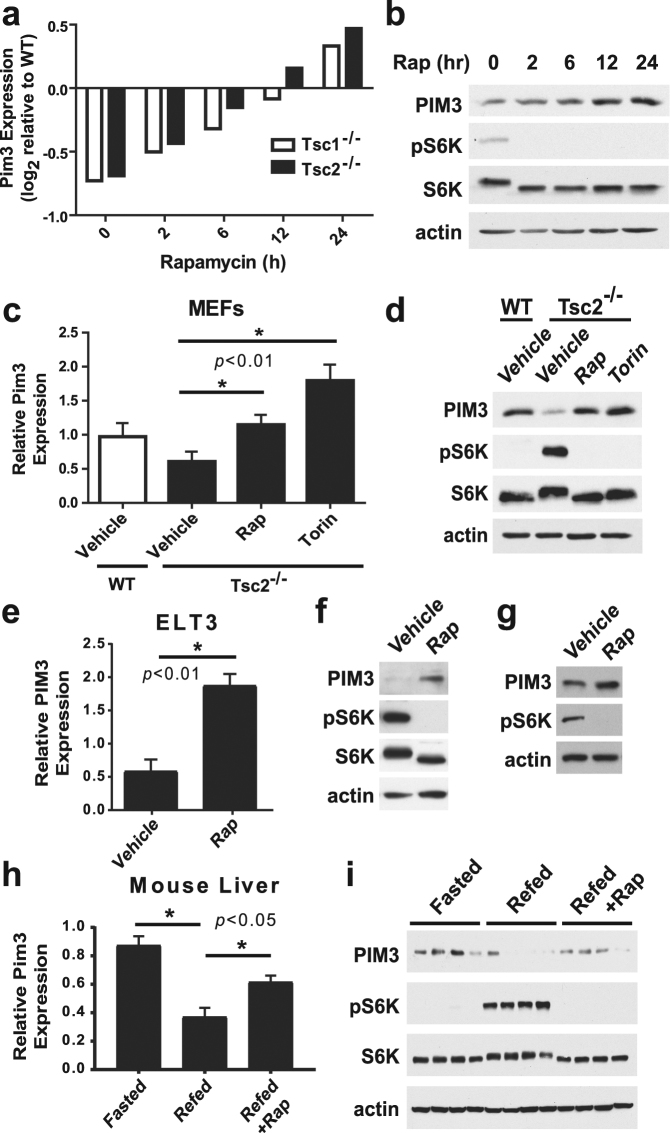
PIM3 is repressed downstream of mTORC1. (**a**) Microarray data for Pim3 expression levels in *Tsc1* ^−/−^ or *Tsc2* ^−/−^ mouse embryonic fibroblasts (MEFs), normalized to wild-type MEFs and treated with a 24-hour time-course of rapamycin (20 nM). (**b**) Protein levels of PIM3 in mTORC1-activated *Tsc2* ^−/−^ MEFs starved and treated with a 24-hour time-course of rapamycin (Rap, 20 nM). (**c**) mRNA and (**d**) protein levels of PIM3 in mTORC1-activated (*Tsc2* ^−/−^) MEFs compared with *Tsc2*-wild-type MEFs. Cells were starved and treated overnight with vehicle, rapamycin (Rap, 20 nM) or torin (250 nM). N = 4 for qPCR; data are shown as mean ± s.e.m. (**e**) mRNA and (**f**) protein levels of PIM3 in TSC2^−/−^ rat ELT3 cells. Cells were starved and treated overnight with vehicle or rapamycin (Rap, 20 nM). N = 3 for qPCR; data are shown as mean ± s.e.m. (**g**) Protein levels of PIM3 in wild-type MEFs starved and treated overnight with vehicle or rapamycin (Rap, 20 nM). (**h**) mRNA and (**i**) protein levels of PIM3 in mouse liver samples fasted during the day and then treated with vehicle or rapamycin (Rap, 10 mg/kg) for 30 minutes before refeeding. N = 4 for qPCR; data are shown as mean ± s.e.m. *Statistical significance determined by a two-tailed *t*-test. Gels shown are cropped; full-length gels are presented in Supplementary Figure 1.

Through analysis of gene expression array data from *Tsc1* ^−/−^ and *Tsc2* ^−/−^ mouse embryonic fibroblasts (MEFs), which have constitutive mTORC1 activation, Pim3 transcript levels were found to be repressed relative to untreated wild-type MEFs and induced in a time-dependent manner over 24 hours of treatment with the mTORC1 inhibitor rapamycin (Fig. 1a). PIM3 protein levels increased with similar kinetics, peaking with prolonged rapamycin treatments of 12 or 24 hours (Fig. 1b). We confirmed that both PIM3 mRNA and protein levels were decreased in *Tsc2* ^−/−^ MEFs and found that mTORC1 inhibition with either rapamycin or the mTOR kinase domain inhibitor Torin1 restored PIM3 expression to wild-type levels (Fig. 1c,d). The ability of rapamycin to induce PIM3 mRNA and protein levels was also observed in ELT3 cells, a cellular model of TSC derived from a *TSC2* ^−/−^ uterine leiomyoma arising in the Eker rat (Fig. 1e,f)^28^. Even suppression of the low basal mTORC1 signaling in wild-type MEFs with rapamycin led to a further increase in PIM3 expression, demonstrating that this effect is not specific to genetic settings with loss of TSC2 (Fig. 1g).

*In vivo*, mTORC1 signaling is highly sensitive to feeding status, especially in the liver, being repressed during fasting and acutely activated upon feeding. Thus, to determine whether physiological control of mTORC1 signaling influenced PIM3 expression, PIM3 mRNA and protein levels were measured in liver samples from mice that had been fasted or fed with or without rapamycin pretreatment. PIM3 transcript and protein levels were highest in the fasted state and were strongly suppressed upon feeding, coincident with activation of mTORC1 signaling, indicated by phosphorylation of its direct downstream target S6K1 (Fig. 1h,i). Importantly, the feeding-induced suppression of PIM3 was largely dependent on mTORC1 activation, as PIM3 mRNA and protein levels remained elevated in liver samples from mice treated with rapamycin just prior to feeding.

**Figure 2:**
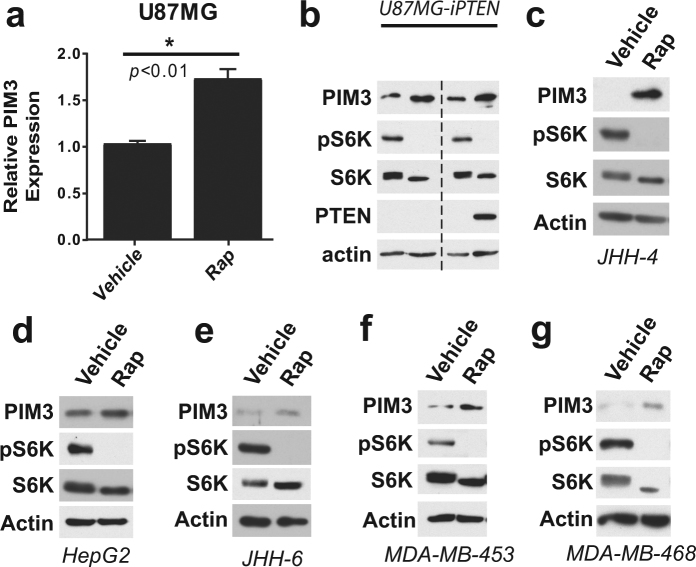
PIM3 repression by mTORC1 is observed in a variety of human cancer settings. (**a**) mRNA levels of PIM3 in U87MG glioblastoma cells. Cells were starved and treated overnight with vehicle or rapamycin (Rap, 20 nM). N = 3 for qPCR; data are shown as mean ± s.e.m. (**b**) PIM3 protein levels in U87MG cells with doxycycline-inducible PTEN (U87MG-iPTEN). Cells were starved and treated overnight with vehicle, rapamycin (Rap, 20 nM), or doxycycline (Dox, 1 µg/mL). (**c**–**e**) PIM3 protein levels in hepatocellular carcinoma cell lines JHH-4, HepG2, and JHH-6. Cells were treated overnight with vehicle or rapamycin (Rap, 20 nM) in full serum. (**f,g**) PIM3 protein levels in breast cancer cells lines MDA-MB-453 and MDA-MB-468. Cells were starved and treated overnight with vehicle or rapamycin (Rap, 20 nM). *Statistical significance determined by a two-tailed *t*-test. Gels shown are cropped; full-length gels are presented in Supplementary Figure 2.

To ascertain the broader applicability of these findings, we determined the effects of rapamycin on PIM3 in human cancer cell lines that exhibit growth factor-independent mTORC1 activation and varying levels of PIM3 (Fig. 2). In human glioblastoma cells (U87MG) with activated mTORC1 signaling downstream of PTEN loss, PIM3 transcript levels were increased upon mTORC1 inhibition (Fig. 2a). In U87MG cells expressing a doxycycline-inducible PTEN (U87MG-iPTEN), repression of mTORC1 signaling either with rapamycin or PTEN re-expression via doxycycline treatment led to elevated PIM3 (Fig. 2b). PIM3 levels were also increased to varying degrees by rapamycin treatment in the hepatocellular carcinoma (HCC) lines JHH-4, Hep-G2 and JHH-6 (Fig. 2c–e), and the breast cancer cell lines MDA-MB-453 and MDA-MB-468 (Fig. 2f,g). Therefore, mTORC1 signaling suppresses PIM3 expression in a variety of mammalian settings, resulting in PIM3 induction by rapamycin.

### A survey of mTORC1-regulated transcription factors identifies SREBP1 and 2 as upstream of PIM3

**Figure 3:**
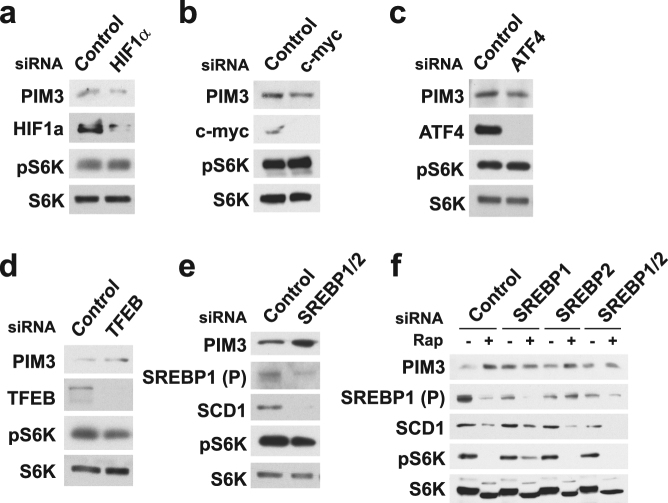
Identification of the mTORC1 effectors SREBP1 and 2 as being upstream of PIM3 regulation. PIM3 protein levels upon transient siRNA knockdown of a panel of transcription factors downstream of mTORC1: (**a**) hypoxia-inducible factor 1 alpha (HIF1α); (**b**) c-myc; (**c**) activating transcription factor 4 (ATF4); (**d**) transcription factor EB (TFEB); or (**e**) sterol regulatory element-binding proteins 1 and 2 (SREBP1/2). Full-length precursor (P) form of SREBP1 is shown. *Tsc2* ^−/−^ MEFs were transfected for 72 hours and starved overnight for the final 16 hours before lysis. (**f**) PIM3 protein levels in *Tsc2* ^−/−^ MEFs upon transient siRNA knockdown of SREBP1, SREBP2, or SREBP1 and 2 (SREBP1/2). Full-length precursor (P) form of SREBP1 is shown. Cells were transfected for 72 hours and starved for the final 16 hours with vehicle or rapamycin (Rap, 20 nM) treatment before lysis. Gels shown are cropped; full-length gels are presented in Supplementary Figure 3.

Due to the nature of our original transcriptional profiling experiment and the timescale over which PIM3 is induced by rapamycin, we hypothesized that a transcription factor downstream of mTORC1 influences PIM3 expression. We therefore tested siRNAs targeting a panel of transcription factors established to be downstream of mTORC1 for effects on PIM3 levels in *Tsc2* ^−/−^ MEFs, including hypoxia-inducible factor 1 alpha (HIF1α), c-myc, activating transcription factor 4 (ATF4), transcription factor EB (TFEB), and sterol regulatory element-binding proteins 1 and 2 (SREBP1/2) (Fig. 3). None of these knockdowns affected mTORC1 signaling. Interestingly, only siRNA-mediated knockdown of SREBP1 and 2 resulted in a substantial increase in PIM3 levels (Fig. 3e). The effect of SREBP knockdown was similar to that observed with rapamycin treatment, and knockdown of both SREBP1 and 2 blocked further induction by rapamycin (Fig. 3f).

**Figure 4:**
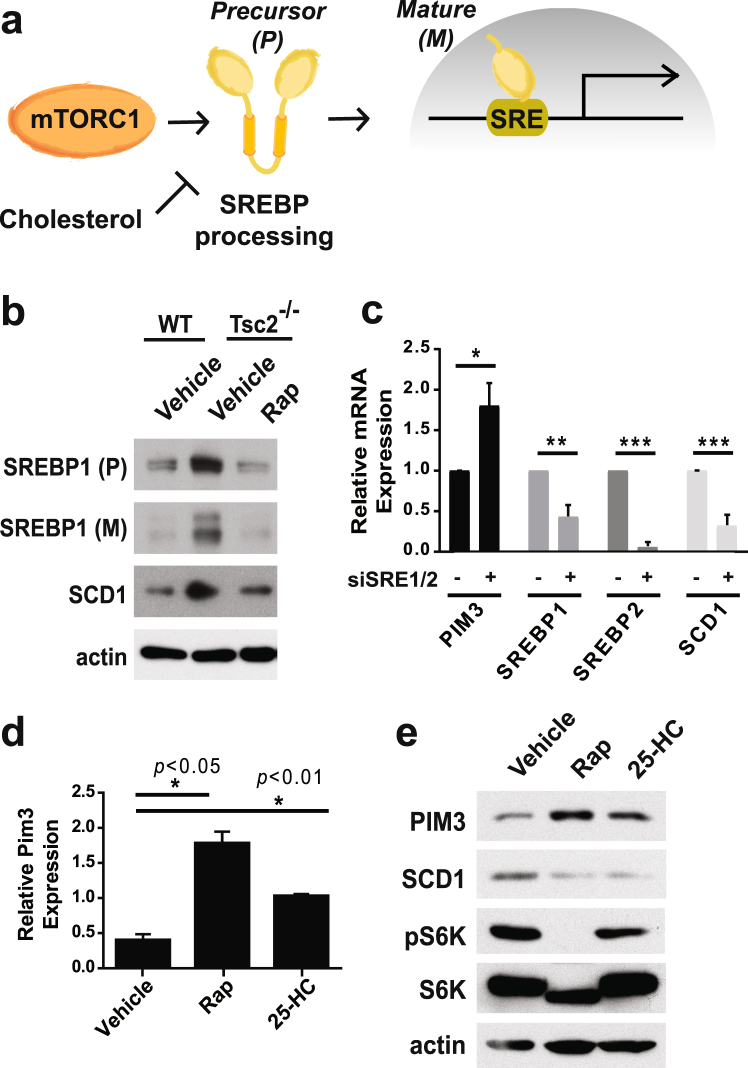
PIM3 is induced upon inhibition of SREBP1 and 2. (**a**) Schematic of SREBP stimulation by mTORC1 and its subsequent induction of transcription from sterol response elements (SREs) in target genes. (**b**) Protein levels of full-length precursor SREBP1 (SREBP1 (P)), mature SREBP1 (SREBP1 (M)), and its target SCD1 in *Tsc2* wild-type or *Tsc2* ^−/−^ MEFs treated with vehicle or rapamycin (Rap, 20 nM). Cells were starved and treated overnight for 16 hours. (**c**) mRNA expression levels of PIM3, SREBP1, SREBP2, and SCD1 upon SREBP1/2 double knockdown with siRNA. Cells were transfected for 72 hours and starved overnight for the final 16 hours. N = 3 for qPCR; data are shown as mean ± s.e.m., *p < 0.5, **p < 0.01, ***p < 0.005. (**d**) PIM3 mRNA and (**e**) protein levels in *Tsc2* ^−/−^ MEFs starved and treated for 16 hours with vehicle, rapamycin (Rap, 20 nM) or 25-hydroxycholesterol (25-HC, 1 μg/mL). N = 3 for qPCR; data are shown as mean ± s.e.m. *Statistical significance determined by a two-tailed *t*-test. Gels shown are cropped; full-length gels are presented in Supplementary Figure 4.

Full-length SREBP is retained as an inactive precursor form on the membrane of the endoplasmic reticulum (ER), and mTORC1 signaling promotes its proteolytic processing at the Golgi and subsequent nuclear localization of its mature form, which binds to sterol regulatory elements (SREs) in the promoters of the genes that it induces (Fig. 4a)^23^. Therefore, in *Tsc2* ^−/−^ MEFs, mature SREBP protein levels are elevated, resulting in increased transcription of numerous SREBP target genes^18^ including its canonical target SCD1 (Fig. 4b). Treatment with rapamycin decreased both the precursor and mature forms of SREBP1 and lowered expression of SCD1 to levels similar to wild-type MEFs. siRNA-mediated knockdown of SREBP1 and 2 increased PIM3 transcript levels, concurrent with a decrease in the mRNA levels of SREBP1, SREBP2, and SCD1 (Fig. 4c). Independent of mTORC1 signaling, SREBP processing and activation is sensitive to intracellular sterol levels^29^. Interestingly, treatment of *Tsc2* ^−/−^ cells with 25-hydroxycholesterol (25-HC), which, like rapamycin, strongly inhibits SREBP processing and expression of SCD1, resulted in increased PIM3 mRNA and protein levels despite sustained mTORC1 activity (Fig. 4d,e). Thus, inhibition of SREBP with either siRNAs or sterols overrides the mTORC1-mediated suppression of PIM3 expression.

### miR-33, an intronic microRNA within the SREBP loci, targets PIM3 downstream of mTORC1

**Figure 5:**
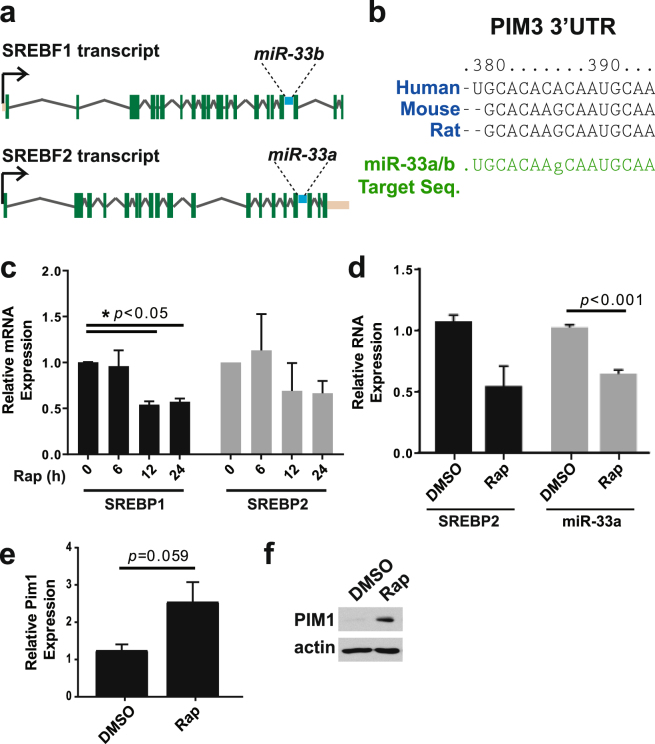
An SREBP-intronic microRNA, miR-33, targets PIM3 expression downstream of mTORC1. (**a**) SREBP1 and SREBP2 contain intronic sequences for miR-33b and miR-33a, respectively^74^. (**b**) The PIM3 3′UTR contains a conserved miR-33 target sequence (Targetscan)^33^. (**c**) SREBP1 and SREBP2 mRNA transcript levels in *Tsc2* ^−/−^ MEFs starved and treated with vehicle or rapamycin (Rap, 20 nM) for a 24-hour time-course. N = 3, data are shown as mean ± s.e.m. (**d**) SREBP2 and miR-33a transcript levels in *Tsc2* ^−/−^ MEFs starved and treated overnight (16 hr) with vehicle or rapamycin (Rap, 20 nM). N = 3, data are shown as mean ± s.e.m. (**e**) mRNA and (**f**) protein levels of PIM1 in mTORC1-activated (*Tsc2* ^−/−^) MEFs. Cells were starved and treated overnight with vehicle or rapamycin (Rap, 20 nM). N = 3 for qPCR; data are shown as mean ± s.e.m. Gels shown are cropped; full-length gels are presented in Supplementary Figure 5.

SREBP1 and 2 are primarily characterized as activators of gene transcription, although a few examples of transcriptional repression have been described^30,31^. However, there are no discernible SREs in the PIM3 promoter, indicating that SREBP1 and 2 are unlikely to directly repress PIM3 expression. Interestingly, the SREBP1 and 2 loci (gene names *SREBF1* and *2*) contain intronic microRNAs that are expressed upon transcription of SREBP (Fig. 5a)^27^. These microRNAs encoded by the SREBP1 and 2 loci, respectively miR-33b and miR-33a, differ by just two nucleotides and thus widely target the same set of transcripts^32^. It should be noted that while humans express both miR-33 forms, mice only possess miR-33a^27^. Importantly, the PIM3 3′UTR contains a consensus target sequence for miR-33 that is conserved throughout mammals, including in mouse, rat, and human (Fig. 5b), all species where rapamycin induces PIM3 expression (Figs 1, 2)^33^. Furthermore, a recent study found that miR-33 does indeed target the PIM3 transcript^34^. While mTORC1 signaling promotes the processing of SREBP1 and 2, it also induces transcription of the SREBP loci due to autoregulation from SREs present in both the SREBP1 and 2 promoters^18,23,35,36^. We confirmed that the transcription of SREBP1 and 2 is sensitive to prolonged rapamycin treatments of 12 to 24 hours (Fig. 5c). Importantly, this decrease in SREBP transcript levels resulted in corresponding decreases in miR-33a levels upon rapamycin treatment (Fig. 5d).

The PIM kinase PIM1 is highly homologous to PIM3^37^, and its mRNA transcript has also been shown to be a target of miR-33a^38^. Therefore, we investigated its expression levels in *Tsc2* ^−/−^ MEFs upon mTORC1 inhibition with rapamycin treatment. Consistent with this shared regulatory feature with PIM3, PIM1 mRNA and protein levels were induced by rapamycin (Fig. 5e,f).

**Figure 6:**
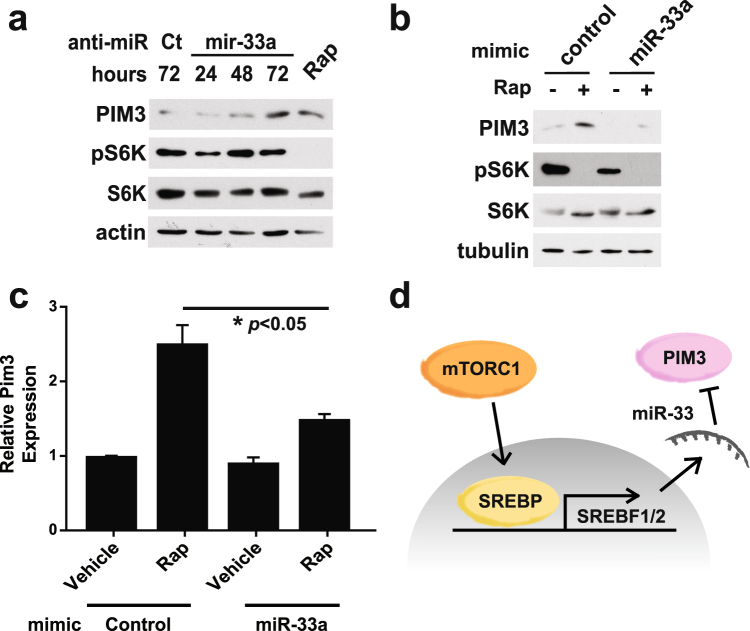
Manipulation of miR-33a levels directly affects PIM3 expression. (**a**) PIM3 protein levels in *Tsc2* ^−/−^ MEFs over a time-course of treatment with a miR-33a inhibitor. Cells were transfected for 24, 48, or 72 hours with the final 16 hours starved overnight with vehicle or rapamycin treatment (Rap, 20 nM). (**b**) PIM3 protein and (**c**) mRNA levels in *Tsc2* ^−/−^ MEFs upon treatment with a miR-33a mimic. Cells were reverse transfected for 24 hours and starved overnight with vehicle or rapamycin treatment (Rap, 20 nM) for the final 16 hours. N = 2 for qPCR; data are shown as mean ± s.e.m. (**d**) Proposed model of the results. *Statistical significance determined by a two-tailed *t*-test for qPCR data. Gels shown are cropped; full-length gels are presented in Supplementary Figure 5.

To determine whether mTORC1 signaling suppresses PIM3 expression through the induction of miR-33, we tested the effects of both an anti-miR inhibiting miR-33a and a miR-33a mimic in *Tsc2* ^−/−^ MEFs. Introduction of the anti-miR targeting miR-33a induced a time-dependent increase in PIM3 expression, similar to the de-repression observed with rapamycin treatment (Fig. 6a). Conversely, a mimic of miR-33a decreased PIM3 levels and attenuated the ability of rapamycin to induce PIM3 at both the mRNA and protein levels (Fig. 6b,c). It is interesting to note that while we observed concomitant changes in PIM3 mRNA and protein levels with the miR-33a mimic, a previous study found that miR-33 mimics reduced PIM3 protein without detectable effects on its transcript levels^34^. As microRNAs are well known to affect both mRNA stability and translation^39^, it is possible that the inhibitory mechanism of miR-33 on the PIM3 transcript could vary at different timepoints and between different settings. Collectively, our data suggest that mTORC1 signaling suppresses PIM3 through the induction of SREBP transcription and a corresponding increase in miR-33, which blocks PIM3 expression (Fig. 6d).

## Discussion

mTORC1 is aberrantly activated in the majority of human cancers, and increasing evidence has highlighted the vital role of sustained mTORC1 signaling in resistance to targeted therapies of upstream pathways^40,41^. While a number of studies have indicated that mTORC1 inhibition is necessary for therapeutic response to oncogene-targeted therapies, it is also recognized that mTORC1 inhibition alone is generally not sufficient for a robust anti-tumor response in most settings^2^. mTORC1 inhibitors induce autophagy, which can have pro-survival effects on tumor cells^42^, and also relieve feedback inhibition of receptor tyrosine kinase signaling leading to enhanced activation of the pro-survival kinase Akt^43,44^, suggesting cell-survival mechanisms are promoted by mTORC1 inhibition. A more thorough understanding of signal wiring and rewiring upon mTORC1 activation and inhibition could help explain the cytostatic effect of mTORC1 inhibitors and suggest promising candidates for the design of combination therapies. In this study, we demonstrate that a proto-oncogenic kinase, PIM3, is repressed downstream of mTORC1, and that its expression is induced upon treatment with the mTORC1 inhibitor rapamycin. Importantly, this induction of PIM3 expression is also observed under physiological control of mTORC1 in the liver with fasting and feeding. Furthermore, induction of PIM3 by rapamycin was also observed in a variety of human cancer cell lines. Our data suggest an additional mechanism limiting the effectiveness of mTORC1 inhibitors as single agent cancer therapies.

There has been increasing interest in the PIM kinases in cancer due to their role as pro-survival kinases. Indeed, they phosphorylate a consensus sequence highly similar to that preferred by AGC family kinases such as Akt and S6K, and PIM kinases have overlapping substrates with these kinases that contribute to their pro-survival and pro-growth role in cells^43,45,46^. The PIM kinases are unique in that they are constitutively active and have a short half-life, such that their levels are proportional to their cellular activity^47^. While PIM levels are regulated primarily at the transcriptional and protein stability levels^48–51^, there are also reports implicating miRNAs as key regulators of PIM expression^34,38,52^. Consistent with these reports, we find evidence that both PIM3 and PIM1 expression are repressed downstream of mTORC1 via its induction of SREBP1 and 2 transcriptional activity and a subsequent increase in miR-33 levels. Interestingly, the inhibition of these kinases downstream of mTORC1 could reflect a novel negative feedback mechanism, as the PIM kinases have been suggested to induce mTORC1 signaling via TSC2 and PRAS40 phosphorylation in some settings^53,54^. Furthermore, a recent study found that PIM1 is upregulated in breast cancer cells that are resistant to the PI3K inhibitor BYL719 and that all three PIM kinases are capable of sustaining mTORC1 signaling and cell proliferation in the presence of the inhibitor in these resistant cells^55^. Whether upregulation of the PIM kinases contributes to the limited effectiveness of, or resistance to, mTOR inhibitors in some settings is an interesting area for future investigation.

Several recent studies have found that mTORC1 inhibitors can cause pronounced changes in cellular miRNAs^56,57^. This is partly due to mTORC1-mediated repression of the microRNA processing enzyme DROSHA^58^, which leads to a general increase in miRNA expression upon mTORC1 inhibition; however, a subset of microRNAs were found to be decreased upon rapamycin treatment. The ability of mTORC1 to modulate the expression of miRNAs provides new insights into the full molecular effects of mTORC1 inhibitors, and alterations in the expression of miR-33 may play a key role in the response to these inhibitors in some settings. A recent study suggested that miR-33a inhibition might decrease sensitivity to cisplatin treatment in HCC^59^. Perhaps this effect is partly due to increased levels of PIM3 in this setting, and other uncharacterized miR-33 targets might contribute to the pro-survival effect of miR-33 inhibition. However, other studies have suggested that high levels of miR-33 might be detrimental to cancer therapy^60,61^. These conflicting findings highlight that the role of miR-33 in cancer and therapeutic responses remains to be fully elucidated.

Consistent with their expression requiring transcriptional induction of the SREBP1/2 loci, the majority of miR-33 targets that have been characterized to date are involved in fatty acid and cholesterol metabolism^27,32,62^. Given that the PIM kinases are believed to have a high degree of functional redundancy, it is interesting to note that PIM1 appears to stabilize the cholesterol transporter ABCA1, which is also a canonical miR-33 target, providing a rationale for PIM targeting by miR-33^63^. While many canonical PIM targets are involved in cell survival, recent work has also implicated them in various metabolic pathways, including glycolysis and mitochondrial biogenesis^64–67^. Furthermore, transgenic mice with human PIM3 expression in the liver exhibited increased lipid droplet accumulation when challenged with a carcinogen^68^, indicating that the PIM kinases may have as-yet undefined roles in lipid regulation, and providing further rationale for their regulation downstream of SREBP and miR-33. The metabolic consequences of decreased miR-33 and subsequent increase in PIM3 and PIM1, therefore, might also influence the cellular and systemic responses to mTOR inhibitors in cancer and other disease settings. Interestingly, chronic and complete inhibition of mTORC1 in the liver has been found to enhance carcinogen-induced hepatocellular carcinoma^69^, an effect also observed with liver-specific overexpression of PIM3^68^.

Our data here expand the functional consequences of mTORC1 activation and inhibition, demonstrating that its regulation of the SREBP transcription factors, thereby affecting levels of the intron-encoded miR-33, influences cellular proteins and processes beyond the program of lipid synthesis directly regulated by SREBP. In future studies, it will be important to identify additional targets of miR-33 that are induced by mTORC1 inhibitors and their contribution to the response of cells, tissues, and systems to these inhibitors, in both physiological and pathological settings.

## Methods

### Cell culture and RNAi

The immortalized litter-mate derived pair of *Tsc2* ^+/+^ and *Tsc2* ^−/−^ (both p53^−/−^) MEFs were provided by Dr. D.J. Kwiatkowski (Brigham and Women’s Hospital), and maintained in Dulbecco’s Modified Eagle Medium (DMEM; VWR, Radnor, PA, USA) with 4.5 g/L glucose containing 10% fetal bovine serum (FBS)^70,71^. MDA-MB-453 and MDA-MB-468 cells were obtained from the American Type Culture Collection (ATCC, Manassas, VA, USA) and maintained in RPMI-1640 with 10% FBS at 37 °C and 5% CO_2_. *TSC2* ^−/−^ ELT3 cells (Eker Rat uterine leiomyoma/myosarcoma tumor-derived) were provided by Dr. C. Walker (Texas A&M University), and maintained in DF8 medium (50% DMEM, 50% F-12, 1.2 g/ml NaHCO3, 1.6 muM FeSO4, 50 nM sodium selenite, 25 mug/ml insulin, 200 nM hydrocortisone, 10 mg/ml transferrin, 1 nM triiodothyronine, 10 muU/ml vasopressin, 10 nM cholesterol, 10 ng/ml epidermal growth factor) containing 15% FBS^28,72^. U87MG-iPTEN cells were maintained in the presence of geneticin (G418, 0.4 mg/mL, Sigma-Aldrich, St. Louis, MO, USA) in DMEM with 4.5 g/L glucose containing 10% FBS, and were developed in the laboratory of M.M. Georgescu (MD Anderson Cancer Center)^73^. JHH-4, JHH-6, and HepG2 cells were obtained from Novartis Institutes for BioMedical Research (Cambridge, MA), and maintained in DMEM with 4.5 g/L glucose containing 10% FBS. All siRNA-mediated knockdown experiments were carried out with ON-TARGET-plus SMARTpool siRNAs (30 nM, GE Dharmacon, Lafayette, CO, USA). Cells were transfected using Lipofectamine RNAiMax (Invitrogen, Carlsbad, CA, USA) according to the manufacturer’s protocol for reverse transfection. Rapamycin (553210, Calbiochem, San Diego, CA, USA), and Torin1 (4247, R&D Systems, Minneapolis, MN, USA) were used to inhibit mTOR; 25-hydroxycholesterol (H1015, Sigma-Aldrich) was used to inhibit SREBP.

### Immunoblotting

Cells were lysed in ice-cold NP-40 lysis buffer (40 nM HEPES [pH 7.4], 400 nM NaCl, 1 mM EDTA [pH 8.0], 1% NP-40 [CA-630, Sigma-Aldrich], 5% glycerol, 10 mM pyrophosphate, 10 mM β-glycerophosphate, 50 mM NaF, 0.5 mM orthovanadate) containing protease inhibitor cocktail (P8340, Sigma-Aldrich) and 1 µM microcystin-LR (ALX-350–012-C500, Enzo Life Sciences, Farmingdale, NY, USA). Lysates were clarified by centrifugation (20,000 × g for 15 min at 4 °C) and protein concentrations were determined with Bradford assay (Bio-Rad) prior to normalization. The following antibodies were used for detection of proteins transferred to immobilon-P PVDF membranes after SDS-PAGE: β-actin (A5316, Sigma-Aldrich), tubulin (T5168, Sigma-Aldrich), TFEB (A303-673A, Bethyl, Montgomery, TX, USA), HIF1-α (10006421, Cayman Chemical, Ann Arbor, MI, USA), SREBP1 (for human samples; sc-8984, Santa Cruz), SREBP1 (for mouse samples; 557036, BD Biosciences), PIM1 (sc-13513, Santa Cruz). All other antibodies were obtained from Cell Signaling Technologies (Danvers, MA, USA): PIM3 (4165), SCD (2438), P-S6K1-T389 (9234), Total-S6K1 (2708), ATF4 (11815), and c-Myc (13987).

### mRNA and miRNA expression analysis

Microarray data were obtained and analyzed as previously described^18^. RNA was isolated using the RNeasy Mini Kit (Qiagen, Valencia, CA, USA). Complementary DNA was synthesized using the Superscript III First Strand Synthesis System (Invitrogen) and after dilution in nuclease-free water was quantified using SYBR-Green for quantitative reverse transcription polymerase chain reaction (Bio-Rad CFX Connect Real-Time System). Each condition was run in triplicate and normalized to *RPLP0* (*m36b4*), or *Actin*. Primer sequences for mouse mRNAs were as follows: *Pim3* (F 5′-CGACATCAAGGACGAGAACC-3′, R 5′-CTCCTCATCCTGCTCAAAGG-3′); *Srebf1* (F 5′-TAGATGGTGGCTGCTGAGTG-3′, R 5′-GATCAAAGAGGAGCCAGTGC-3′); *Srebf2* (F 5′-GGATCCTCCCAAAGAAGGAG-3′, R 5′-TTCCTCAGAACGCCAGACTT-3′); *Scd* (F 5′-CTGACCTGAAAGCCGAGAAG-3′, R 5′-GCGTTGAGCACCAGAGTGTA-3′); *Pim1* (F 5′-CGACATCAAGGACGAGAACA-3′, R 5′-GTAGCGATGGTAGCGAATCC-3′). Primer sequences for human mRNAs were as follows: *PIM3* (F 5′-AAGCTCATCGACTTCGGTTC-3′, R 5′-AGGATCTCCTCGTCCTGCTC-3′).

Small RNAs for miRNA measurement were isolated using the miRNeasy Mini Kit (Qiagen). Complementary DNA was synthesized using the miScript II RT Kit (Qiagen) and quantified using SYBR-Green for qRT-PCR (Bio-Rad CFX Connect Real-Time System). Each condition was run in triplicate and normalized to RNU6. Primer assays were purchased from Qiagen (hsa-miR-33_1 [MS00003304], hsa-miR-33b_2 [MS00007819], hsa-RNU6-2_11 [MS00033740]).

### miRNA inhibitors and mimics

For miR-33a inhibition, miRCURY LNA power microRNA inhibitors were purchased from Exiqon (Vedbaek, Denmark): inhibitor control A (199006-002) and hsa-miR-33a-5p (4102039-102). Cells were transfected at a final concentration of 10 nM inhibitor with RNAiMax Lipofectamine for 24, 48, or 72 hours and starved for the final 16 hours before lysis. For miR-33a mimics, mirVana miRNA Mimics were purchased from Thermo Fisher Scientific (Waltham, MA, USA): control mimic #1 (4464058) and miR-33a-5p mimic (MC12410). Cells were reverse transfected following the manufacturer’s protocol at a final concentration of 30 nM with RNAiMax Lipofectamine for 24 hours, and either starved (MEFs) or left in full serum (U87MG) for the final 16 hours, plus vehicle or rapamycin (20 nM).

### Mouse fasting and refeeding experiment

Twelve C57BL/6 J male mice (aged 8 weeks) were fasted for 8–10 hours during the light cycle and either euthanized (n = 4) or administered either vehicle (5% Tween-80, 5% PEG-400 in 1x PBS; n = 4) or rapamycin (10 mg/kg; n = 4) via intraperitoneal injection 30 minutes prior to refeeding normal chow for 4 hours during the dark cycle, followed by euthanization. Liver lysates were prepared in RIPA buffer (150 mM Sodium Chloride, 1% IGEPAL, 0.5% sodium deoxycholate, 0.1% SDS and 50 mM Tris [pH 8]). mRNA was processed with the RNeasy Mini Kit (Qiagen), and complementary DNA was synthesized with the iScript cDNA Kit (Bio-Rad, Hercules, CA, USA). mRNA transcript levels were quantified by qRT-PCR as described above.

### Ethics Approval

All animal experiments in this study were approved by the Harvard Medical Area’s Institutional Animal Care and Use Committee and were performed in accordance with the approved guidelines for animal experimentation at Harvard Medical School.

### Statistical Analysis

All qRT-PCR data were analyzed with GraphPad Prism (La Jolla, CA, USA). *P-*values were calculated by an unpaired two-tailed Student’s *t*-test, where appropriate.

### Data Availability

All data generated during this study are included in this published article and its Supplementary Information files. Raw qRT-PCR data from multiple independent experiments, represented in graphical form in the manuscript, are available from the corresponding author on request.

## Electronic supplementary material

Supplementary Figures
