# Supplementary material for: mTORC1 suppresses PIM3 expression via miR-33 encoded by the SREBP loci
Source: Sci Rep. 2017 Nov 23;7:16112. doi: 10.1038/s41598-017-16398-y (PMC5701013; doi:10.1038/s41598-017-16398-y)

**mTORC1 suppresses PIM3 expression via miR-33 encoded by the SREBP loci**

Ilana Kelsey^1^, Marie Zbinden^1^, Vanessa Byles^1^, Margaret Torrence^1^, and Brendan D. Manning^1*^

^1^Department of Genetics and Complex Diseases, Harvard T.H. Chan School of Public Health, Boston, MA, USA

*Correspondence to: bmanning@hsph.harvard.edu

**Supplementary information**

**Supplemental Figures**

**Supplementary Figure 1**. Uncropped blots for panels shown in **(A)** Figure 1B, **(B)** 1D, **(C)** 1F (note: there is a prominent cross-reacting band below 30 kD with the PIM3 antibody in these rat cells, which is not detected in mouse or human cells), **(D)** 1G, and **(E)** 1I.


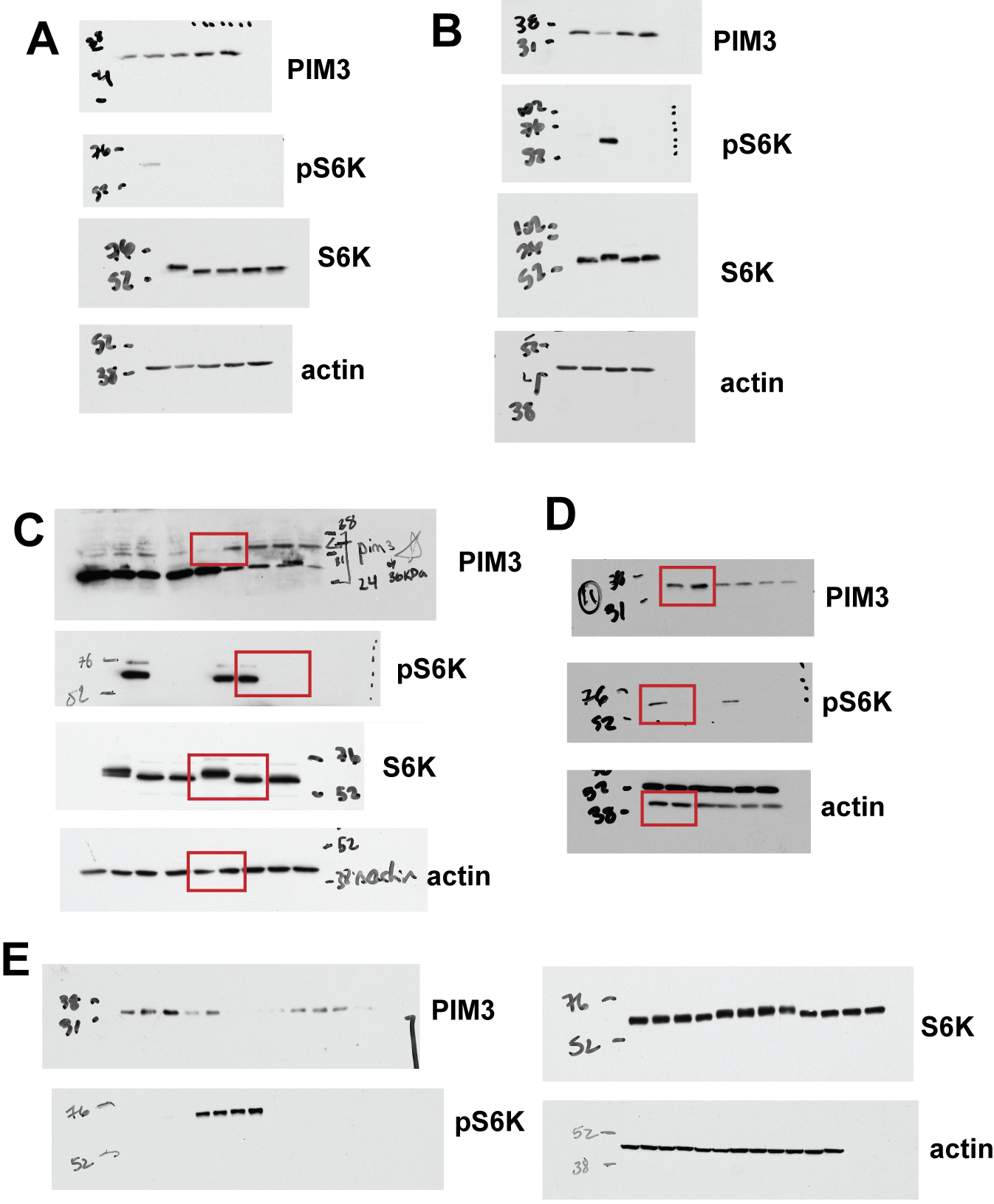


**Supplementary Figure 2**. Uncropped blots for panels shown in **(A)** Figure 2B, **(B)** 2C, **(C)** 2D, **(D)** 2E, **(E)** 2F, **(F)** 2G.


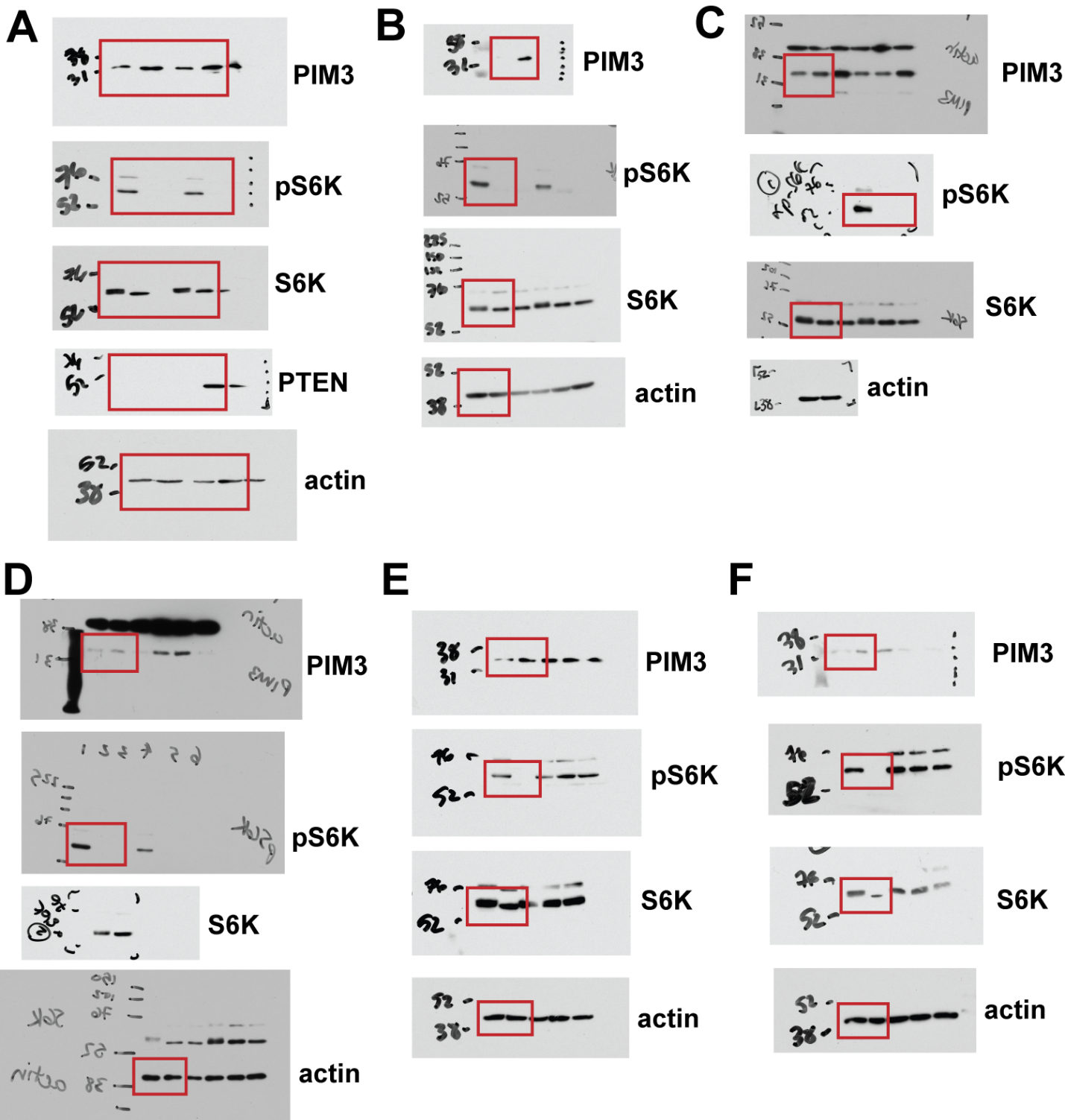


**Supplementary Figure 3**. Uncropped blots for panels shown in **(A)** Figure 3A, **(B)** 3B, **(C)** 3C, **(D)** 3D, **(E)** 3E, and **(F)** 3F.


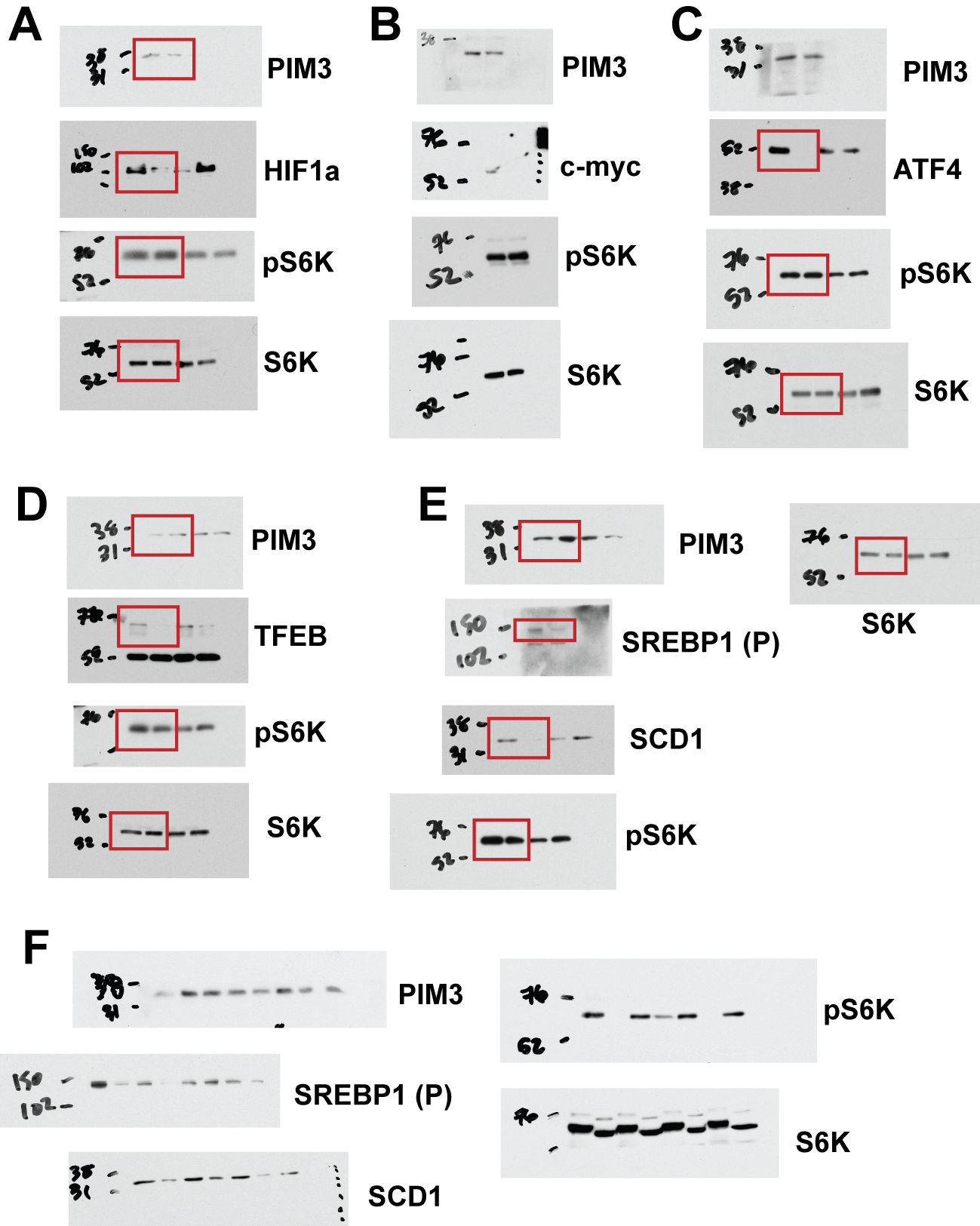


**Supplementary Figure 4**. Uncropped blots for panels shown in **(A)** Figure 4B and **(B)** 4E.


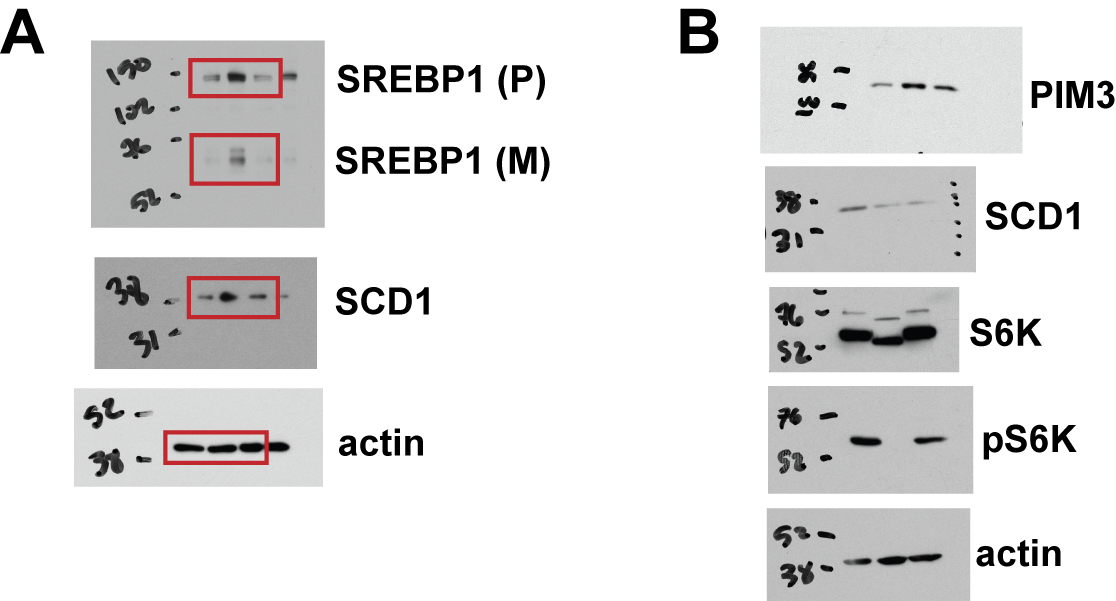


**Supplementary Figure 5**. Uncropped blots for panels shown in **(A)** Figure 5F, **(B)** Figure 6A, and **(C)** Figure 6B.


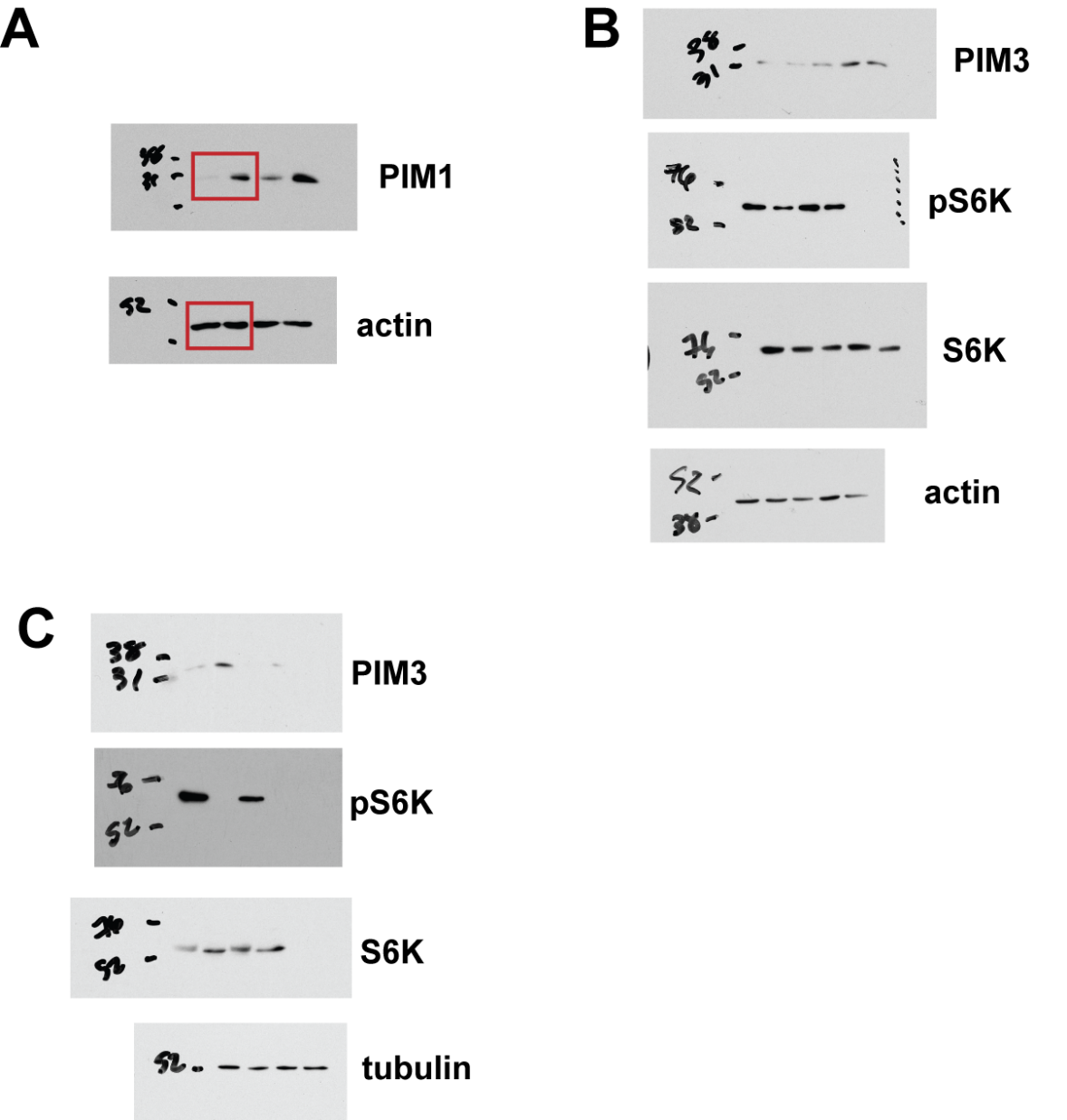

Supplement: Supplementary file 1 — Supplementary Figures [file 41598_2017_16398_MOESM1_ESM.doc]
